# Relative harms and merits of postoperative radiation following conservative surgery for low risk breast cancer

**DOI:** 10.1038/sj.bjc.6600005

**Published:** 2002-01-21

**Authors:** K Holli, R Saaristo, J Isola, H Joensuu, M Hakama

**Affiliations:** University Hospital, Palliative Medicine, PO Box 2000, 33521 Tampere, Finland

## Abstract

*British Journal of Cancer* (2002) **86**, 310–311. DOI: 10.1038/sj/bjc/6600005 www.bjcancer.com

© 2002 The Cancer Research Campaign

## Sir

Buchholz and Singletary raise the following issues in their commentary to our study (Holli *et al*, 2001):

- the breast preservation rate should not have been reported as an endpoint as it was not predetermined by the protocol and, therefore, it is subject to bias
- no aesthetic outcomes were reported
- loco-regional recurrences are likely to be associated with distant metastases and an increased risk of death. Hence, longer follow-up after salvage therapy is needed.

The mastectomy rate following conservative surgery depends on a number of factors, such as the relative size of the recurrent tumour with respect to the breast size, location of the tumour, availability of breast reconstruction, skill and experience of the surgeon, and patient preference. Therefore, we preferred not to give strict guidelines in the study protocol for the type of breast surgery used following recurrence, nor did we select the breast preservation rate as a study endpoint. Had the breast preservation rate been predetermined as an endpoint, the attitudes of the clinicians might have influenced the choice of the type of surgery more than in the present design, where no emphasis was given on the issue prospectively. Hence, it is possible that our study design may have been even less biased than after predetermining the breast preservation rate as an endpoint.

Cosmetic outcome was not reported in our article, but we did study it by asking the subjective opinions of the patient and the physician. The results on the cosmetic outcome are shown in Table 1

**Table 1:**
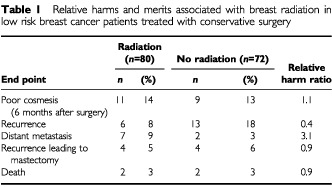
Relative harms and merits associated with breast radiation in low risk breast cancer patients treated with conservative surgery

. We considered the cosmetic result poor only when both the patient and physician regarded the result as such. There was no difference between the study arms in the cosmetic outcome 6 months following surgery. The cosmetic result following removal of the recurrent tumour was not assessed formally.

It is generally accepted that loco-regional recurrences are associated with an increased risk for distant metastases and death. However, the data is scanty in the subset of very low risk patients. We intentionally selected a very low risk subgroup for the study, and when looking at the 6-year survival rates it is evident that we truly succeeded in determining such a group. Nevertheless, as we emphasized in our article, the risk for local relapse is quite high (18% in our study) even in this very low risk group when radiotherapy is omitted. We fully agree with Buchholz and Singletary that a long follow-up time is needed in this low risk group, because the tumours, in particular, are likely to have a slow cell proliferation rate, and both local and distant metastases may need a long time to appear. We plan to perform a new analysis after 10-year follow-up of the patient cohort. Seven patients in the post-operative radiation group and two in the non-radiation group have recurred distally, and we apologize for the incorrect numbers in Figure 2 of our previous paper (Holli *et al*, 2001).

The main message from the study is that radiotherapy decreases the risk for local relapse even in low risk patients after conservative breast surgery. However, it remains to be confirmed that radiotherapy decreases the rate of mastectomies in this carefully selected and closely followed subgroup of patients, where the option for reexcision and radiotherapy may still be available when radiotherapy has not been given primarily. In general our recommendation is to give radiotherapy to all patients with invasive breast cancer after conservative surgery to avoid local relapses, and we agree that omission of radiotherapy should be done only in the context of a clinical study.
